# Bioenergy and Biomass Utilization

**DOI:** 10.1155/2015/857568

**Published:** 2015-06-11

**Authors:** Guangli Cao, Dexun Zou, Ximing Zhang, Lei Zhao, Guojun Xie

**Affiliations:** ^1^School of Life Science and Technology, Harbin Institute of Technology, Harbin 150001, China; ^2^College of Chemical Engineering, Beijing University of Chemical Technology, Beijing 100029, China; ^3^Laboratory of Renewable Resources Engineering, Purdue University, West Lafayette, IN 47907-2032, USA; ^4^State Key Laboratory of Urban Water Resource and Environment, Harbin Institute of Technology, Harbin 150090, China; ^5^Advanced Water Management Centre, The University of Queensland, Brisbane, QLD 4072, Australia

Bioenergy is of increasing interest as a renewable, environmentally friendly alternative to energy derived from fossil fuels. Through a variety of processes, biomass can be converted to solid, liquid, or gaseous biofuels. However, various challenges persist to the maintenance and development of biomass-to-energy utilization. This special issue focuses on the most recent advances in the frontier research of bioenergy and biomass utilization and features six selected papers with high quality.

The paper titled “Nanofibrillated Cellulose and Copper Nanoparticles Embedded in Polyvinyl Alcohol Films for Antimicrobial Applications” by T. Zhong et al. develops a method to produce hybrids of TEMPO nanofibrillated cellulose (TNFC) and copper nanoparticles. The hybrid material is embedded in polyvinyl alcohol thermoplastic resin and the films are produced using a solvent casting method. The authors evaluate the films in terms of its morphology and thermal and antimicrobial properties and prove that TNFC-copper nanoparticles as antimicrobial nanofillers are valuable for PVA applications.

The paper titled “Response of Arbuscular Mycorrhizal Fungi to Hydrologic Gradients in the Rhizosphere of* Phragmites australis* (Cav.) Trin ex. Steudel Growing in the Sun Island Wetland” by L. Wang et al. assesses the variations of hydrologic gradients in the relationships among AM fungi, reed, and rhizospheric microorganisms. The authors discover water content in soil and reed growth parameters are both positively associated with AM fungi colonization, but only the positive correlations between reed biomass parameters and the colonization could be expected, or both the host plant biomass and the AM fungi could be beneficial. This study could shed light on the mechanisms inside AM fungi-reed symbioses and would be referred to for optimizing the combined phytorhizoremediation.

The paper titled “Production by Tobacco Transplastomic Plants of Recombinant Fungal and Bacterial Cell-Wall Degrading Enzymes to Be Used for Cellulosic Biomass Saccharification” by L. Paolo et al. presents work on saccharification of plant biomass. The authors express in tobacco chloroplasts microbial genes encoding five cellulases and a polygalacturonase. Leaf extracts containing the recombinant enzymes show the ability to degrade various cell-wall components under different conditions. In addition, a thermostable xylanase is also tested in combination with a cellulase and a polygalacturonase to study the cumulative effect on the depolymerization of a complex plant substrate. Results demonstrate the feasibility of using transplastomic tobacco leaf extracts to convert cell-wall polysaccharides into reducing sugars, fulfilling a major prerequisite of large scale availability of a variety of cell-wall degrading enzymes for biofuel industry.

The paper titled “Enzymatic Saccharification of Lignocellulosic Residues by Cellulases Obtained from Solid State Fermentation Using* Trichoderma viride*” by T. Sartori et al. presents an approach that uses cellulolytic complex produced by* Trichoderma viride* in solid state fermentation to hydrolyze lignocellulosic residues. This produced enzyme shows viability in comparison with the commercial cellulase enzyme. The synthesis of cellulases by microorganisms from lignocellulosic residues is a process of great interest, representing the search for renewable sources to replace the fossil energetic matrix.

The paper titled “Improving Biomethane Production and Mass Bioconversion of Corn Stover Anaerobic Digestion by Adding NaOH Pretreatment and Trace Elements” by C. Liu et al. describes an approach to improve biomethane production from corn stover. The authors use NaOH pretreatment and trace elements supplementation as a strategy to enhance the biodegradability of corn stover for biomethane production. The approach is compared with only NaOH-pretreated and untreated corn stover, and the paper highlights the supplementation of trace elements.

The paper titled “Biochemical Modulation of Lipid Pathway in Microalgae* Dunaliella* sp. for Biodiesel Production,” by A. F. Talebi et al., explores the use of myoinositol to modulate LP and biodiesel quality. Inclusion of myoinositol in the media indeed improves the total lipid accumulation and biodiesel quality parameters, and this work highlights the fact that the biochemical modulation strategies should be progressively considered in the hope of finding more efficient and economically feasible strategies leading to more viable production systems.

